# Supplementary material for: Characterizing cell-type spatial relationships across length scales in spatially resolved omics data
Source: Nat Commun. 2025 Jan 3;16:350. doi: 10.1038/s41467-024-55700-1 (PMC11699133; doi:10.1038/s41467-024-55700-1)
Supplement: Supplementary file 3 — Supplementary Software 1 [file 41467_2024_55700_MOESM3_ESM.zip › CRAWDAD/plots/supplementary/S7_cluster_exp_PKHL_annot.pdf]

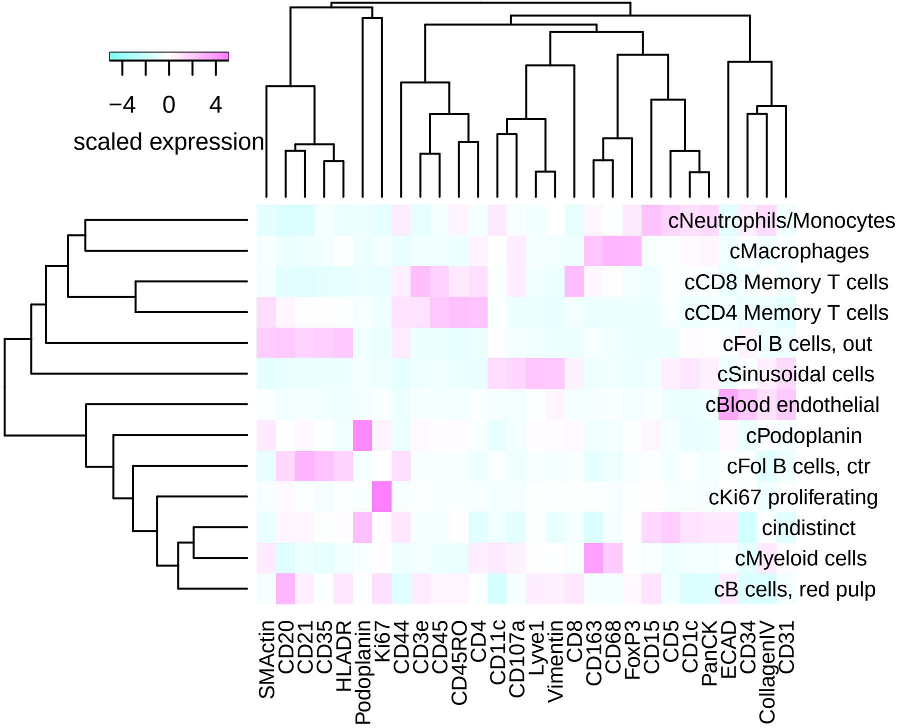

−4
0
4
scaled expression
cNeutrophils/Monocytes
cMacrophages
cCD8 Memory T cells
cCD4 Memory T cells
cFol B cells, out
cSinusoidal cells
cBlood endothelial
cPodoplanin
cFol B cells, ctr
cKi67 proliferating
cindistinct
cMyeloid cells
cB cells, red pulp
SMActin
CD20
CD21
CD35
HLADR
Podoplanin
Ki67
CD44
CD3e
CD45
CD45RO
CD4
CD11c
CD107a
Lyve1
Vimentin
CD8
CD163
CD68
FoxP3
CD15
CD5
CD1c
PanCK
ECAD
CD34
CollagenIV
CD31
